# (Cost-)effectiveness and implementation of a combined lifestyle intervention for outpatients with severe mental illness (GOAL!): a hybrid quasi-experimental study protocol

**DOI:** 10.1186/s12888-024-06216-x

**Published:** 2024-11-14

**Authors:** C. R. Noortman-van Meteren, M. M. E. van Schothorst, N. M. den Bleijker, B. Braakhuis-Keuning, W. M. H. Houwert-Zuidema, T. A. M. J. van Amelsvoort, J. Deenik

**Affiliations:** 1 Science Department, GGz Centraal, Amersfoort, the Netherlands; 2 Mental Health and Neuroscience Research Institute, Faculty of Health, Medicine and Life Sciences, Maastricht University, Maastricht, the Netherlands; 3 Department of Psychiatry, UMC Utrecht Brain Center, University Medical Center Utrecht, Utrecht University, Utrecht, the Netherlands

**Keywords:** combined lifestyle intervention, outpatients, severe mental illness, lifestyle behaviors, health outcomes, effectiveness, implementation, cost-effectiveness, peer support workers, allied health professionals

## Abstract

**Background:**

People with severe mental illness (SMI) face not only impaired mental health, but also a greater risk of physical comorbidities and a shorter life expectancy compared to the general population. A poor lifestyle plays a substantial role in this disparity. *Combined Lifestyle Interventions* targeting multiple lifestyle behaviors can improve mental and physical health, and quality of life. However, there is currently no appropriate structural support for people with SMI in outpatient care in the Netherlands. The *Combined Lifestyle Intervention for Outpatients with SMI (GOAL!)* is developed to address this gap. This study examines the (cost-)effectiveness and implementation of GOAL!.

**Methods:**

In a type 1 hybrid quasi-experimental study with a mixed-method matched design, GOAL! participants (*N* = 50) are compared to people receiving care as usual (*N* = 50). The GOAL! program includes group and individual sessions, given by allied health professionals, over a period of two years. The first year starts with a 3-month intensive course on physical activity and nutrition, followed by 9 months of aftercare covering various lifestyle topics tailored to the group’s needs. There is close collaboration with local stakeholders to facilitate transfer to the community setting. The second year focuses on maintaining established activities in one’s daily living environment. Our primary outcome will be the change in physical activity, comparing GOAL! participants to those receiving care as usual. Secondary outcomes are changes in other lifestyle behaviors, physical health, mental well-being, and healthcare and societal costs. Additionally, achieving lifestyle-related goals, adverse effects, and barriers and facilitators to implementation are examined. Measurements are obtained at start (T0), and after 3 (T1), 12 (T2) and 24 months (T3).

**Discussion:**

This study investigates the effects of GOAL! on lifestyle behaviors, health outcomes, implementation factors and cost-effectiveness after two years, aiming to offer valuable insights into the effectiveness and implementation outcomes of lifestyle interventions for outpatients with SMI.

**Trial registration:**

: ClinicalTrials.gov (Identifier: NCT05600205). Prospectively registered on October 26, 2022.

## Background

People with a severe mental illness (SMI) have a greater prevalence of physical conditions compared to the general population [1–3]. SMI mainly concerns people with psychotic, personality, and major mood disorders who require long-term care. They have up to two times higher risk of developing cardiometabolic diseases, irrespective of their diagnosis [4]. This multimorbidity leads to elevated healthcare utilization [2], a heightened risk of hospitalization [3], and a shorter life expectancy of up to 15 years compared to the general population [5–8]. In addition, people with multimorbidity have increased difficulties in daily living, psychiatric symptoms, and cognitive functioning [9]. The prevalence of this multimorbidity and reduced life expectancy appears to be increasing, highlighting the urgent need for targeted interventions to improve their overall health [5, 10–12].

Lifestyle behaviors play a substantial role in the health of people with SMI [4, 11, 13]. They often have unhealthy lifestyle behaviors, such as low physical activity levels, poor nutritional intake, disturbed sleeping patterns, and high smoking rates [13–18]. Because lifestyle behaviors are modifiable, there is great potential to improve the mental and physical health of people with SMI by targeting lifestyle changes within their treatment. Studies have shown that lifestyle interventions can improve not only lifestyle factors, such as physical activity [14, 19, 20], nutrition [21], sleep [15], and smoking cessation [18, 22], but also physical health [19, 23–25], mental health [19], psychosocial functioning, quality of life [23, 26], and medication use [27]. Long-term (i.e. longer than 12 months) multidisciplinary support focused on multiple lifestyle behaviors seems most effective for intervention success [4, 13]. Additionally, support should focus on both education and behavioral change [16]. The physical and social characteristics of community contexts in which people with SMI reside can also play an important role in promoting or hindering healthy lifestyle behaviors [28]. Interventions addressing the social determinants of health, such as reducing loneliness and isolation, and improving social participation (e.g., employment), may have the greatest potential to reduce early mortality [12]. In summary, long-term multidisciplinary interventions incorporating multiple lifestyle behaviors, behavioral strategies, and considering environmental factors holds promise for achieving sustainable improvements in the health and life expectancy of people with SMI.

There are existing lifestyle interventions that follow this perspective, but they are often inappropriate for people with SMI. In the Netherlands, the *Combined Lifestyle Interventions (CLIs)* target multiple lifestyle behaviors simultaneously and are reimbursed in the national health insurance scheme for everyone with (a high risk of) diabetes or obesity. These CLIs are based on effective *Diabetes Prevention Programs* [29, 30], which are also identified in the literature as the “gold standard” for people with SMI [4]. However, people with SMI are often excluded from CLIs because of their psychopathology, medication use, or lack of motivation [31, 32]. This highlights the lack of tailored support that addresses their increased challenges to lifestyle changes, such as illness-related symptoms, physical comorbidities, stress, and medication side effects [33–36]. For example, the direct and indirect side effects of medication can form a barrier through causing metabolic disturbance, body weight gain, movement disorders, and increased appetite [35, 37–40]. People with SMI, relatives and healthcare practitioners indicate the need for lifestyle interventions for people with SMI in outpatient care, who often lag behind public health progress [4, 13, 23].

To address this gap, the *Combined Lifestyle Intervention for Outpatients with SMI (GOAL!)* was developed. GOAL! provides multidisciplinary support from allied health professionals and peer support workers for two years. In this program, participants receive group-based and individual support on various lifestyle behaviors. GOAL! builds on effective components of existing CLIs, but is tailored to address the needs of people with SMI. The program was co-created with people with lived experience, relatives, peer support workers, healthcare practitioners, CLI experts, healthcare insurers, and community partners (e.g., policy makers of municipalities and community service providers). This resulted in a program with increased individualized support and guidance from professionals with expertise in mental healthcare and related challenges (e.g., medication side effects). To facilitate a personalized transfer to people’s own daily living environment, there is close collaboration with local stakeholders within the community setting, thereby aiming for sustainable lifestyle behavior change.

The aim of this study is to investigate the (cost-)effectiveness and implementation of GOAL!. This provides a comprehensive understanding of the effects of such a lifestyle intervention on lifestyle behaviors, physical health, mental well-being, and healthcare and societal costs in outpatients with SMI. By identifying barriers and facilitators to implementation, we gain insights into underlying mechanisms behind intervention success and failure. These results can inform decision making by stakeholders involved in the implementation of such support for people with SMI within outpatient care.

## Methods

### Design and setting

This is a type 1 hybrid quasi-experimental study with a mixed-method matched design. This design allows for the assessment of the effectiveness of GOAL! on health-related outcomes while collecting and analyzing implementation data [41]. An intervention group (*N* = 50) will be compared with a control group (*N* = 50) who continue to receive care as usual and are matched by demographic characteristics. GOAL! is designed in collaboration with stakeholders in multiple municipalities in the Netherlands where GOAL! will be implemented. To avoid contamination, the control group will be recruited in separate municipalities. Due to the nature of the intervention, and the innovative approach to care of this research, randomization or blinding is not possible. Measurements are obtained at start (T0), and after 3 (T1), 12 (T2), and 24 months (T3).

### Eligibility criteria

People are eligible to participate if they are 18 years or older, diagnosed with SMI, have an abdominal circumference of ≥ 102 cm for men or ≥ 88 cm for women, and have at least one of the other four risk criteria as clustered in the metabolic syndrome: hypertension (systolic ≥ 130 and/or diastolic ≥ 85 mmHg); abnormal triglycerides (≥ 1.7 mmol/l); fasting blood sugar (≥ 5.6 mmol/l); HDL cholesterol (< 1.0 mmol/l for men; <1.3 mmol/l for women); or pharmacological treatment for one of these. People are not eligible if they, or a legal representative, are unable to provide written informed consent, if addiction is the primary diagnosis, or if the severity of the disease does not allow participation at that time (e.g. acute psychosis or suicidality, in consultation with their relevant healthcare practitioner). Additionally, residents of the intervention region are eligible to participate in GOAL!, while residents of the control region are eligible to participate in the control group.

### Recruitment

GOAL! is promoted within mental health organizations in the intervention region through flyers, presentations, and introductory meetings. Healthcare practitioners invite eligible people to participate in GOAL!. Interested participants are referred to a lifestyle coach for an intake session. Upon enrollment, GOAL! participants are encouraged to voluntarily participate in the evaluation. If participants express interest, the lifestyle coach assists in establishing communication with the research team for potential inclusion. GOAL! participants who take part in the evaluation will receive gift vouchers of €5 at T0, €10 at T1, €15 at T2, and €30 at T3.

In collaboration with mental health organizations in the control region, eligible participants are invited by their healthcare practitioners to enroll in the control group. Healthcare practitioners will then facilitate communication for interested participants with the research team for potential inclusion. Participants of the control group will receive gift vouchers of €10 at T0, €20 at T1, €40 at T2, and €50 at T3.

### Sample size

The sample size calculation is based on changes in lifestyle behaviors and health outcomes, for which we used data from a previous study on inpatients in the Netherlands [19]. A sample size of 98 participants is necessary to detect an average effect (f^2^ = 0.15) in a linear regression analysis with 80% power, 95% confidence interval, and six variables in the model. After reaching consensus on feasibility with the collaborating region, it was decided to enroll 50 people to participate in the GOAL! program and recruit an equally sized control group.

### Intervention

**Fig. 1:**
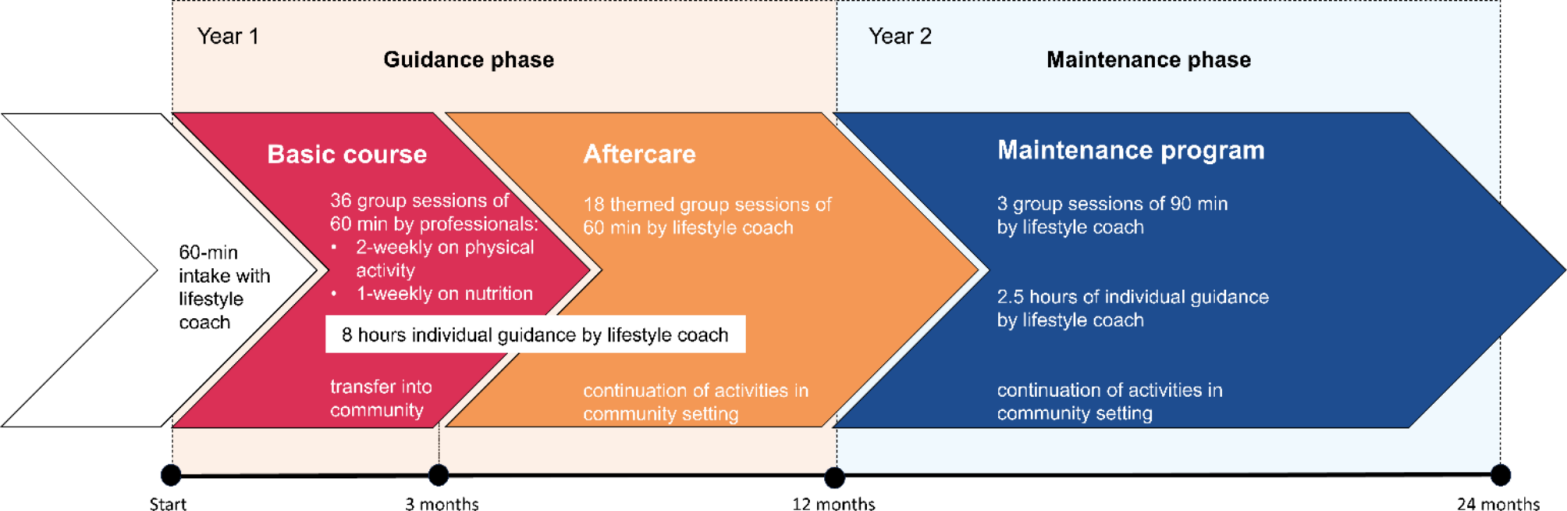
Design of the GOAL! intervention

GOAL! provides multidisciplinary support from registered allied health professionals and peer support workers. In a two-year program, participants receive group-based and individual support on different lifestyle behaviors. The program is divided into the guidance phase (first year), and the maintenance phase (second year) (Fig. 1).

The program starts with an intake session of 60 min conducted by the lifestyle coach, during which lifestyle behaviors, health outcomes, and individual needs are examined. Lifestyle goals are set through shared decision making, a collaborative process proven to enhance effectiveness [42]. In the guidance phase (i.e., year one), the program starts with a 3-month basic course, which encompasses 36 group sessions, each lasting 60 min, guided by allied health professionals. This includes 2-weekly sessions on physical activity (24 sessions in total) and 1-weekly session on nutrition (12 sessions in total). To achieve sustainable lifestyle behavioral change, there is a close collaboration with local stakeholders, thereby facilitating a transfer to the community setting. Thereafter, the aftercare of 9 months starts, with a thematic session of 60 min every two weeks (18 sessions in total) guided by the lifestyle coach about other lifestyle-related topics. These themes can vary based on the needs of the group and include themes such as smoking, sleep, and stress. In the guidance phase, participants receive 8 h of individual support from the lifestyle coach. This coaching can be utilized at the discretion of the participant and is centered around their self-identified lifestyle goals. As the participants’ options, needs and goals evolve, the coaching is adjusted accordingly. Group and individual sessions include theoretical education, interactive elements, assignments, physical activity, and supplementary practical activities (e.g., supermarket visits).

During the maintenance phase (i.e., second year), there are 3 group sessions of 90 min each, and 5 moments of individual support of 30 min each, both guided by the lifestyle coach. Participants are encouraged to continue their activities in the community setting while the lifestyle coach has a key role in communication and connection with community partners as needed. Following each phase, lifestyle coaches assess and evaluate the changes in lifestyle behaviors and health outcomes together with the participants, setting new goals accordingly through shared-decision making.

### Outcome measures

The primary outcome is the change in physical activity between participants of GOAL!, compared to participants who receive care as usual. Secondary outcomes focus on changes in other lifestyle behaviors, physical health, mental well-being, and healthcare and social costs. In addition, the achievement of lifestyle goals, adverse effects, and barriers and facilitators to implementation are examined. Implementation factors are also investigated among healthcare practitioners and stakeholders.

### Measurements

#### Demographic characteristics

At baseline, all participants are asked about their personal characteristics, including gender, nationality, ethnicity, date of birth (for age calculation), marital status, highest level of education, primary source of income, and income level. The diagnosis is obtained from patient records to ensure reliability.

#### Lifestyle behaviors

##### Physical activity and sedentary behavior

The Simple Physical Activity Questionnaire (SIMPAQ) is used to assess physical activity and sedentary behavior. The SIMPAQ consists of 5 items (boxes), averaged over the past 7-day period: time spent in bed overnight (box 1), time sedentary, including napping (box 2), time spent walking (box 3), time spent exercising (box 4) and time spent in incidental activity (box 5). The time spent walking and exercising (boxes 3 and 4) will be combined to calculate the total self-reported moderate-vigorous physical activity time, in hours per week. Alternatively calculating sedentary behavior in hours per day is advised, by subtracting the total non-sedentary behavior (adding boxes 1,3,4 and 5) from 24 h. The SIMPAQ is a reliable and valid tool for assessing physical activity and sedentary behavior in people with SMI [43].

##### Eating behavior

Eating behavior is measured through a Dutch online tool called ‘MijnEetmeter’, a food diary offered on the website of The Nutrition Center of the Netherlands [44]. The relative validity of MijnEetmeter is similar to that of other self-administered apps, although there appears to be more underreporting compared to interviewer-administered 24-h recalls [45]. We chose MijnEetmeter because of its usability for both practical and research purposes. This free tool includes nearly all Dutch food products, enabling users to log their nutritional intake. It calculates daily nutrient intake and consumption of food groups according to the Dutch ‘Wheel of Five’ dietary guidelines [46]. MijnEetmeter provides comprehensive insights into dietary patterns for participants and professionals, facilitating nutritional coaching. Additionally, it offers feedback on dietary behavior, fostering self-management. The tool was initially used in nutrition sessions, but was not found to be sufficiently user-friendly for participants, leading to its discontinuation for practical purposes and its exclusive use for research. The participants were asked to report their dietary intake for the previous day.

##### Sleep

The Scales for Outcomes in Parkinson’s disease sleep (SCOPA-sleep) is used to assess sleep. The SCOPA-sleep examines total scores for nighttime sleep quality (5 items) answered on 4-point Likert scales ranging from 0 (not at all) to 4 (heavily). In addition, the measurement assesses daytime sleepiness (6 items) from never (0) to often (4) [47]. The SCOPA-sleep is a reliable and valid instrument for assessing nighttime and daytime sleep in people with mental disorders [48].

##### Substance use

Substance use is assessed by the Alcohol, Smoking, and Substance Involvement Screening Test Lite (ASSIST-Lite). The ASSIST-Lite examines total scores for the use, frequency, urges, control and worries about the use of tobacco, alcohol, cannabis, stimulants, sedatives, opioids, and psychoactive substances. Questions are asked via Likert scales ranging from 0 (never) to 4 (multiple times a week or daily), and ordinal scales ranging from 0 (no) to 1 (yes). The instrument has been adapted and recognized for use in health and social care settings [49]. Items about tobacco smoking are additionally added to retrospectively calculate the packs per year [50].

#### Physical health outcomes

Weight (kg) and body fat percentage are measured by the Omron BF214 scale [51], with participants wearing clothes but without shoes. Length (m) is requested from the participant and BMI (kg/m²) is calculated. Blood pressure (mmHg) is measured by the Omron M7 Intelli IT blood pressure monitor [52]. The waist circumference (cm) is measured by the SECA 201 [53] measuring tape at the level of the navel in a standing position.

#### Mental health outcomes

##### Psychiatric symptoms

The Brief Symptom Inventory (BSI) is used to assess psychiatric symptoms. The BSI consists of 53 items covering 9 symptom domains of the SCL-90-R, examining scores for 9 symptom dimensions: somatization, obsession-compulsion, interpersonal sensitivity, depression, anxiety, hostility, phobic anxiety, paranoid ideation, and psychoticism. Each item is assessed via a 5-point Likert scale, ranging from 0 (not at all) to 4 (extremely) [54]. The BSI is reliable and its validity is supported across patient groups with various diagnoses in a Dutch sample [55].

##### Quality of life

The Euro-Qol-5D-3 L (EQ-5D-3 L) is used to measure quality of life. The EQ-5D-3 L measures health-related quality of life in 5 dimensions of health: mobility, self-care, daily activities, pain/discomfort, and anxiety/depression, with one item per dimension, scored from 1 (no problems) to 3 (many problems). The Dutch tariff will be used to estimate the index score based on the preferences of the Dutch population [56]. Moreover, participants are asked to indicate their overall health on the day of questionnaire completion on a scale of 0-100, the Visual Analogue Scale [57]. The EQ-5D-3 L is considered a valid instrument for assessing people with SMI [58].

#### Healthcare and societal costs

The Treatment Inventory of Costs in Patients with psychiatric disorders (TIC-P) is used for the evaluation of healthcare utilization, medication use, and productivity loss due to health problems. The TIC-P consists of 14 questions to assess healthcare utilization in the past 3 months (e.g., doctor visits, treatment, and medication use), and 11 questions about productivity losses due to health problems (e.g., hindering paid and unpaid work). The costs are calculated by multiplying the quantity of healthcare utilization and productivity loss by the related costs according to national guidelines. The TIC-P is a feasible and reliable instrument for people with mental health problems [59].

#### Implementation factors

##### Achieving lifestyle goals and adverse effects

Lifestyle coaches continuously monitor personal lifestyle goals and potential adverse effects during the program. Goals are established at intake and are regularly assessed, reported, and modified in individual coaching sessions.

##### Barriers and facilitators

The Measurement Instrument for Determinants of the Innovation (MIDI) is used to assess the barriers and facilitators to the implementation of GOAL!. The questionnaire consists of 4 constructs related to the innovation (7 determinants), the user (11 determinants), the organization (10 determinants), and the socio-political environment (1 determinant). Determinants can consist of one or more items. The total score of a determinant can be a combined measure calculated as the mean of multiple items. Most items are scored on a Likert scale from 1 (totally disagree) to 5 (totally agree). This questionnaire is also assessed in healthcare practitioners and stakeholders. The constructs addressed vary among the different respondent groups. GOAL! participants are asked about the constructs of innovation and user, healthcare practitioners about all the constructs, and stakeholders about the constructs of innovation, organization, and the socio-political environment. The questionnaire is assessed at start (T0) and 24 months (T3). The MIDI is adapted according to the manual to measure the relevant determinants for implementation of GOAL!, to fit the different respondents (MIDI-GOAL! participants, MIDI-healthcare practitioners and MIDI-stakeholders), and the different assessment points (at the start and end). Thus, there are 6 different versions of the MIDI. The MIDI is used to gain insight into the determinants of the innovation, to learn and improve the program from experiences, and to better interpret health outcomes [60].

##### Process evaluation

Attendance at group sessions, use of individual coaching sessions, and dropout are continuously monitored by the health professionals throughout the program. This monitoring allows for better evaluation of effectiveness and implementation by providing important data on participant engagement, adherence, and reasons for dropout. The implementation process of GOAL! is continuously evaluated through experiences from (potential) participants, healthcare practitioners, and stakeholders. Their input and feedback serve as the foundation for developing new strategies for both the GOAL! program itself and its implementation.

#### Cost-effectiveness

The cost-effectiveness of GOAL! is determined through a Health Technology Assessment (HTA), using the outcomes of the TIC-P. To weigh the costs against health-related outcomes, the main outcome is the deterministic incremental cost-effectiveness ratio using the EQ-5D-3 L scores, resulting in the quality-adjusted life years (QALY). For further evaluation, we perform probabilistic sensitivity analyses to account for the uncertainty surrounding the parameters, resulting in cost-effectiveness planes. The QALY is the most commonly used outcome in the economic evaluation of medical interventions and captures the impact of GOAL! on both the quantity and quality of life [61].

**Table 1:** Overview of assessment procedures, measurements and variables

Assessment points	Outcome measures	Measurements	Variables	Lifestyle coaches	Research team	
**Intervention and control group**						
T0	Demographic characteristics	Gender	Categorical		x	
		Nationality	-		x	
		Ethnicity	Categorical		x	
		Date of birth (age)	Years		x	
		Diagnosis	Categorical		x	
		Marital status	Categorical		x	
		Highest level of education	Categorical		x	
		Primary source of income	Ratio		x	
T0, T1, T2, T3	Physical activity and sedentary behavior	Simple Physical Activity Questionnaire (SIMPAQ)	Moderate-vigorous physical activity in min/week and sedentary time per day	x		
	Eating behavior	MijnEetmeter	Daily intake of nutrients and daily consumption according to the Dutch ’Wheel of Five’		x	
	Sleep	Scales for Outcomes in Parkinson’s Disease sleep (SCOPA-sleep)	Total scores for nighttime sleep quality and daytime sleepiness	x		
	Substance use	Alcohol, Smoking and Substance Involvement Screening Test Lite (ASSIST-Lite)	Total scores of tobacco, alcohol, cannabis, stimulants, sedatives and opioids and psychoactive use	x		
	Physical health	Weight	kg	x		
		Abdominal circumference	cm	x		
		BMI	kg/m^2^	x		
		Fat percentage	%	x		
		Blood pressure	mmHg	x		
	Psychiatric symptoms	Brief Symptom Inventory (BSI)	Total scores for 9 symptom dimensions: somatization, obsession-compulsion, interpersonal sensitivity, depression, anxiety, hostility, phobic anxiety, paranoid ideation and psychoticism		x	
	Quality of life	Euro-Qol-5D-3 L (EQ-5D-3 L)	Index score based on the Dutch tariff and VAS score		x	
T0, T3	Care and social costs	Treatment Inventory of Costs in Patients with psychiatric disorders (TIC-P)	Healthcare utilization, medication use, productivity loss and costs (€)		x	
**Intervention group only**						
Ongoing	Lifestyle goals		Goals achieved yes/no, and explanation	x		
Ongoing	Undesirable effects		Undesirable effects yes/no, and explanation	x		
T0, T3	Implementation factors ^a^	Measurement Instrument for Determinants of the Innovation (MIDI)	Facilitators and barriers related to the intervention		x	

^a^ Also assessed at healthcare practitioners and stakeholders involved in the implementation and collaboration of GOAL!

### Data collection and management

All participants receive study information through a visual informed consent, using visual elements to explain the procedures, risks, and benefits of study participation, thereby enhancing understanding and communication. Additionally, they receive a written information letter, after which they are requested to provide permission by signing an informed consent form. Measurements are taken through semi-structured interviews, physical measurements, and online questionnaires at start (T0), and after 3 (T1), 12 (T2) and 24 months (T3).

For GOAL! participants, data collection will be conducted by the lifestyle coach and the research team. As this research follows innovative care, lifestyle coaches assess lifestyle behavior, physical health outcomes, and lifestyle goals at all four assessment points (T0-T3). This is part of the program but will also be utilized for research purposes. Lifestyle coaches will record examination results on hard-copy documents, which will then be transferred and securely stored by the research team. Trained researchers and research assistants administer additional questionnaires and measurements. These include psychiatric symptoms, quality of life, and eating behavior at all four assessment points (T0-T3), and healthcare utilization, somatic illness, work, and the implementation process only at the start and end of the program (T0 and T3). Research assistants undergo structured training by experienced research team members in conducting semi-structured interviews via research and interview protocols.

The research team collects all data from the control group. Lifestyle behaviors, physical health outcomes, psychiatric symptoms, and quality of life are evaluated at all four assessment points (T0-T3), whereas healthcare utilization, somatic illness, and work are evaluated only at the start and end of study participation (T0 and T3).

All surveys in this study are entered in, and facilitated by Castor EDC, a cloud-based research data management platform [62]. At T1 and T2, participants have the option to complete some questionnaires via an online survey, thus shortening or obviating the need for an interview with the research team (Table 1). Furthermore, healthcare practitioners and stakeholders receive an online survey regarding the implementation process at the start and the end of the program (T0 and T3). Their informed consent is obtained online.

Participants’ personal information will only be used for measurement purposes and to share general study results, as specified in the consent form. The data will be pseudonymized, personal information will be stored separately, and both will be accessible only to the research team. Access to the data requires two-factor authentication. Data is stored within the institution for 15 years, following national privacy laws, and will not be publicly released.

If participants in the GOAL! program decide to withdraw, they will be asked to remain in the evaluation for additional analysis. If participants withdraw their consent for the evaluation, any data collected prior to withdrawal will be stored in accordance with the data protocol and Dutch law but will not be used for analysis or publication.

### Statistical analysis

To assess the effect of GOAL! on lifestyle behaviors and health outcomes, data will be analyzed via multiple linear regressions and linear mixed models (LMMs). LMMs allow for testing the effect over time (four measurement points) and accounting for potential clustering (e.g., participant groups). In all analyses, the treatment condition (GOAL! participants vs. control group, coded as 1/0) will be the independent variable, and the outcomes measures will be the dependent variables, with physical activity (in minutes per week) as the primary outcome. All analyses will be adjusted for potential confounders, such as age (continuous), sex (male vs. female, coded as 0/1), key diagnostic subgroups (categorial, added as dummy variables), baseline values (continuous), session adherence (continuous), and changes in medication use (continuous).

When necessary, missing data will be addressed through multiple imputations via the MICE package in R. When constructing the imputation model, we will include the variables used as predictors in our analysis. The number of imputations and iterations will be based on the proportion of missing data in our LMM. Results from imputed datasets will be pooled using Rubin’s rules.

Implementation factors are processed according to the manual of the measurement instrument. Cost-effectiveness is analyzed in a health-technology assessment.

All analyses will be performed with IBM SPSS statistics 25 and R version 4.04 or higher, based on 95% confidence intervals (*p* < 0.05), and if necessary will be corrected for multiple outcomes (Bonferroni correction).

### Study status

Data collection is ongoing at the time of this manuscript submission and recruitment is expected to be completed in the first half of 2025.

## Discussion

This study protocol is designed to investigate the (cost-)effectiveness and implementation of the *Combined Lifestyle Intervention for Outpatients with SMI (GOAL!)* in the Netherlands. *Combined Lifestyle Interventions (CLIs)* are successfully implemented in the Netherlands and showed improvements in lifestyle behaviors changes and health outcomes in the general population, but people with SMI are often excluded from these programs [31, 63]. GOAL! builds on previous successful CLIs, based on the principles of the effective *Diabetes Prevention Programs* [29, 30, 36]. These principles are also recognized as the “gold standard” for people with SMI when their specific needs are considered [4, 36]. GOAL! addresses a gap identified by people with lived experience, relatives and healthcare practitioners by providing a more suitable lifestyle intervention for outpatients with SMI.

Physical activity is the core of the GOAL! program and holds the most substantial evidence for improving overall health among all lifestyle behaviors [13]. Therefore, the primary outcome of this study is the change in physical activity levels. Nevertheless, we assess a variety of lifestyle and health outcomes. In addition, in complex intervention research, such as in this study, it is crucial to examine outcomes that are most valuable for decision making by stakeholders involved in the implementation, beyond those that can be answered with the greatest certainty [64]. In this study we assess not only the effectiveness of the intervention but also its societal impact, working elements, and interactions with the implementation context by evaluating barriers and facilitators to implementation and the cost-effectiveness.

The strengths of this study include the investigation of a lifestyle intervention specifically tailored to the needs of people with SMI, as identified by recent literature [4, 33, 34, 36] and including the perspectives of people with lived experience, relatives, and healthcare professionals. GOAL! provides long-term guidance of various certified professionals with expertise in mental health, who support individuals in overcoming challenges (e.g., motivational barriers, physiological complaints) that may arise during the program. Furthermore, the content of the sessions and coaching is focused on multiple lifestyle behaviors and is tailored to the participants’ options, needs, and goals, with a strong emphasis on transfer to the community setting. This tailored, long-term, and community-based approach enhances the prospects of intervention success.

Second, the intervention of GOAL! and the design of the study are co-created with peer support workers and relatives’ perspective, insurers and community-based organizations (e.g., municipalities, and welfare organizations). Aside from its contribution to relevance and validity, these collaborations will be valuable for potential further implementation and scale-up if GOAL! is shown to be effective.

A third strength of this study is the evaluation of a variety of outcome measures, providing a comprehensive understanding of health changes. Assessing changes not only in physical health but also in mental well-being is certainly of added value in this population. While lifestyle interventions in the general population are strongly focused on weight loss, these interventions can have an effect on several health-related outcomes, which is especially relevant for people with SMI.

Finally, by identifying the facilitators and barriers to implementation, we can optimize both the program and its implementation process. This effectiveness-implementation type 1 hybrid design is a notable strength, as it facilitates the linkage between effectiveness and implementation which is currently highly needed in this field [65]. It provides a comprehensive understanding of the underlying mechanisms behind intervention success and failure. This approach enhances external validity and supports decision making about further implementation, thereby increasing the practical impact of the findings [41].

Several limitations should be considered within the protocol of this study. First, it is challenging to include people with SMI in intervention research because of illness and healthcare-related complexities. People with SMI often face motivational barriers, self-stigma, psychological complaints, or medication side effects, potentially leading to low enrollment, low adherence, and high dropout rates [33, 34, 66]. Furthermore, healthcare practitioners may encounter inconsistencies in defining who meets the criteria for severe mental illness, organizational barriers (e.g., changes in work practices), and stigma (e.g., negative beliefs and attitudes towards people with SMI, leading to the exclusion of potential participants) [67, 68], which can lead to selection bias.

Second, participants are asked to answer many questions at each measurement point, which can be perceived as burdensome. Some questionnaires may also be difficult to answer or may include sensitive topics. To address these issues, questions can be answered face-to-face or via an online questionnaire, measurements can be spread over multiple sessions, and participants are allowed to skip questions.

Third, for GOAL! participants, half of the questionnaires are administered by the lifestyle coach and the other half by the research team, leading to variability in the data for the outcome measures. However, this is minimized by using as many validated instruments as possible and standardizing measurements and training and consultation throughout the process.

Lastly, the implementation of GOAL! takes place in a select region of the Netherlands, which may limit the generalizability of the findings. In addition, because the control group is recruited in other regions, various contextual factors may influence outcomes. However, this approach helps prevent contamination, and by evaluating the implementation and considering these contextual factors, we aim to gain insights into context-specific barriers, facilitators and processes. This understanding will assist in the interpretation of the outcomes and potential implementation of GOAL! in other regions.

The results of this study will provide a comprehensive understanding of the effectiveness and implementation of GOAL!, serving as essential input for informed decision-making regarding the future integration of lifestyle interventions into outpatient care for people with SMI.

## Data Availability

No datasets were generated or analysed during the current study.

## Abbreviations

ASSIST-Lite: Alcohol, Smoking and Substance Involvement Screening Test Lite
BSI: Brief Symptom Inventory
CLIs: combined lifestyle interventions
EQ-5D-3 L: Euro-Qol-5D-3 L
GOAL!: Combined Lifestyle Intervention for Outpatients with severe mental illness
HTA: Health Technology Assessment
LMMs: linear mixed models
MIDI: Measurement Instrument for Determinants of Innovation
SCOPA-sleep: Scales for Outcomes in Parkinson’s disease sleep
SIMPAQ: Simple Physical Activity Questionnaire
SMI: severe mental illness
TIC-P: Treatment Inventory of Costs in Patients with psychiatric disorders
QALY: quality-adjusted life years
